# Bunyamwera Virus Infection of *Wolbachia*-Carrying *Aedes aegypti* Mosquitoes Reduces *Wolbachia* Density

**DOI:** 10.3390/v16081336

**Published:** 2024-08-21

**Authors:** Daniella A. Lefteri, Stephanie M. Rainey, Shivan M. Murdochy, Steven P. Sinkins

**Affiliations:** MRC-University of Glasgow-Centre for Virus Research, Garscube Campus, University of Glasgow, Glasgow G61 1QH, UK; daniella.lefteri@glasgow.ac.uk (D.A.L.) shivan.murdochy@glasgow.ac.uk (S.M.M.)

**Keywords:** mosquitoes, *Wolbachia*, Bunyamwera, arboviruses, *Aedes*, viruses

## Abstract

*Wolbachia* symbionts introduced into *Aedes* mosquitoes provide a highly effective dengue virus transmission control strategy, increasingly utilised in many countries in an attempt to reduce disease burden. Whilst highly effective against dengue and other positive-sense RNA viruses, it remains unclear how effective *Wolbachia* is against negative-sense RNA viruses. Therefore, the effect of *Wolbachia* on Bunyamwera virus (BUNV) infection in *Aedes aegypti* was investigated using *w*Mel and *w*AlbB, two strains currently used in *Wolbachia* releases for dengue control, as well as *w*Au, a strain that typically persists at a high density and is an extremely efficient blocker of positive-sense viruses. *Wolbachia* was found to reduce BUNV infection in vitro but not in vivo. Instead, BUNV caused significant impacts on density of all three *Wolbachia* strains following infection of *Ae. aegypti* mosquitoes. The ability of *Wolbachia* to successfully persist within the mosquito and block virus transmission is partially dependent on its intracellular density. However, reduction in *Wolbachia* density was not observed in offspring of infected mothers. This could be due in part to a lack of transovarial transmission of BUNV observed. The results highlight the importance of understanding the complex interactions between multiple arboviruses, mosquitoes and *Wolbachia* in natural environments, the impact this can have on maintaining protection against diseases, and the necessity for monitoring *Wolbachia* prevalence at release sites.

## 1. Introduction

The maternally transmitted bacterial endosymbiont *Wolbachia*, which naturally occurs in many arthropod species, spreads itself through populations via reproductive manipulation. This can include cytoplasmic incompatibility (CI), where *Wolbachia*-positive males mating with *Wolbachia*-negative females results in significant reductions in egg hatch. *Wolbachia* does not occur naturally in *Aedes aegypti* mosquitoes but different strains have instead been successfully introduced following lab transfer, resulting in stable *Wolbachia-*carrying mosquito lines [1,2]. These *Wolbachia*-carrying lines have been demonstrated to efficiently block several clinically important positive-sense single-stranded RNA (+ssRNA) arboviruses within the *Aedes aegypti* mosquito, such as flaviviruses and alphaviruses including chikungunya virus (CHIKV) and dengue virus (DENV) [3,4,5,6]. Different types of release programmes for dengue control, including population replacement or suppression strategies, have been conducted in several countries, with high success rates for replacement using strains *w*AlbB in Malaysia [5,7] and *w*Mel in Indonesia and Brazil [6,8,9,10].

Whilst the effectiveness of *Wolbachia* to inhibit arbovirus infections has been demonstrated repeatedly, the mechanisms by which this is achieved are incompletely understood and there is emerging evidence that different mechanisms can be responsible for blocking virus infection between *Wolbachia* strains. The *Wolbachia w*Mel strain causes virus blocking within the mosquito by causing perturbations in various cellular pathways including lipid transport [11]; cyclodextrins such as 2-hydroxypropyl-β-cyclodextrin (2HPCD), which can reverse lipid accumulation defects in cells with disrupted cholesterol homeostasis, rescue infection of Flaviviridae such as ZIKV and DENV in *w*Mel- and *w*AlbB-carrying cell lines, but not *w*Au-carrying cells [11,12]. Furthermore, rescuing these perturbations in *w*Mel and *w*AlbB has no effect on alphavirus replication [11], further supporting the hypothesis that multiple mechanisms are involved. *Wolbachia* density is also an important factor in terms of ensuring efficient virus blocking. In fact, certain environmental conditions such as high cyclic heat patterns have been found to have an adverse effect on the density of certain *Wolbachia* strains [13,14]. This adverse effect on density correlated with a reduction in *Wolbachia*-mediated DENV blocking [14,15].

Whilst *Wolbachia* strains have been demonstrated to efficiently block the majority of +ssRNA arboviruses within the mosquito, their effect on negative-sense single-stranded RNA (-ssRNA) arboviruses has been inconclusive. Mosquito borne-ssRNA viruses mainly belong to the order of Bunyavirales within the genera of Orthobunyaviruses such as La Cross Virus (LACV) and California encephalitis virus (CEV) and Phleboviruses such as Rift Valley fever virus (RVFV). These viruses can also be passed vertically through transovarial transmission [16].

A previous study investigating the effect of *w*Stri on LACV and vesicular stomatitis virus found no blocking of either virus by *w*Stri in *Ae. albopictus* cells [17]. Previous -ssRNA virus research, however, has focused on *Wolbachia* and insect-specific viruses (ISVs). For example, one study has demonstrated no effect on Phasi Charoen-like Bunyavirus by *Wolbachia,* whilst another study observed an enhancement of *Aedes* anphevirus (AeAv) viral titres by *Wolbachia* in cells [18,19]. Whilst these viruses may belong to the same family and genera as some pathogenic -ssRNA viruses, they may not always reflect the same interactions as arboviruses, as ISVs are not transmitted to vertebrates and therefore exhibit different lifecycles.

Differences between +ssRNA and -ssRNA viruses could explain why there may be differences in terms of blocking efficiency between the two groups by *Wolbachia*. Unlike +ssRNA viruses, -ssRNA viruses of the Bunyavirales order are made up of a tri-segmented genome: S, M, and L encoding the nucleocapsid, envelope glycoprotein, and polymerase protein, respectively. These segments are known to reassort, causing an increase in genetic and phenotypic diversity when multiple viruses of the same genus are co-circulating in a population. Due to this, they exhibit different replication cycles where -ssRNA viruses require an RNA-dependent RNA polymerase in order to generate a complementary mRNA strand first [20].

Bunyamwera virus (BUNV), which is a commonly used model virus for studying Bunyavirales viruses, was therefore used in this study to investigate the potential of *Wolbachia* to block its infection. BUNV is an Orthobunyavirus which, alongside other viruses of the same genus, has been found to cause disease in both humans and livestock, posing a potential threat to global health as well as food security. BUNV, whilst likely to be under-surveilled, is believed to be endemic in several African countries [21]. Whilst the transmission patterns of BUNV are yet to be fully characterised, it is believed to be transmitted by *Aedes aegypti* mosquitoes [21,22]. It is currently unknown whether the BUNV used in this study is transmitted between mosquitoes through transovarial transmission. Within the Bunyamwera serogroup, only two viruses have been tested for their ability to be transmitted through transovarial transmission: Cache Valley virus (CVV) [23] and Northway virus (NORV) [24]. Both of these studies observed a very low rate of transovarial transmission.

There is a lack of comprehensive studies on *Wolbachia* interaction with -ssRNA viruses such as BUNV. Therefore, this study sought to determine whether *Wolbachia* can block BUNV infection in cells and in *Ae. aegypti* mosquitoes. This is important as -ssRNA viruses constitute a major global health concern that remains largely understudied.

## 2. Materials and Methods

### 2.1. Mosquito Rearing

*Aedes aegypti* mosquito colonies were maintained in standard 27 °C and 70% relative humidity conditions with a 12 h light/dark cycle. All mosquito lines used have been described and characterised previously [12,25,26]. Briefly, lines were established in 2017 as described by Ant et al. (2018) and have been maintained as colonies [25]. Lines are routinely checked for *Wolbachia* density and show steady levels of *Wolbachia* with little fluctuation under standard conditions.

*Wolbachia*-free lines consist of the original line which *Wolbachia* was transferred into, ensuring all lines retain the same genetic background. Mosquito females were blood fed using a 37 °C Hemotek artificial blood-feeding system (Hemotek, Great Harwood, UK) using human blood donated by the Scottish Blood Bank. Damp filter paper was used as egg cones (Grade 1 filter paper, Whatman plc, GE Healthcare, Hatfield, UK) and was added to cages post mating to allow females to oviposition. Egg cones were collected and desiccated for 5–10 days before being hatched in water containing 1 g/L bovine liver powder (MP Biomedicals, Santa Ana, CA, USA). When pupae formed, these were picked and placed in small water-filled containers and left to emerge into BugDorm mosquito cages. Larvae were fed tropical fish food pellets (Tetramin, Tetra, Melle, Germany). All adult mosquitoes were fed a 10% sucrose solution.

### 2.2. Cell Lines

Aa23 (*Aedes albopictus*) cells naturally infected with *Wolbachia* were either cleared (with tetracycline) or transinfected with *w*Mel or *w*Au strains introduced from *Drosophila simulans* STCP lines as described previously [11,27]. Cells were grown in 25 cm^2^ flasks at 28 °C in Schneider’s Drosophila media (Pan Bioscience, UK) containing 10% heat-inactivated FBS (Thermo Fisher, Altrincham, UK).

Baby Hamster Kidney (BHK-21s) cells were grown at 37 °C with 5% CO2 in Glasgow MEM (GMEM) media supplemented with 5% FBS, 1% Penicillin/streptomycin and 10% Tryptose Phosphate Buffer (Thermo Fisher, Altrincham, UK). Cells were split twice a week at a 1:10 ratio. Cells were frozen with DMSO and kept at −196 °C for long-term storage.

### 2.3. Virus and Plaque Assays

Semliki Forest Virus 4 (SFV4) and Bunyamwera wild-type (BUNV WT) (provided by Alain Kohl, a primary stock created from a viral clone described previously [28]) stocks were generated from plasmids containing infectious cDNA (icDNA) sequences by electroporation into BHK-21 cells to generate infectious virus with two pulses at 250 V for 0.8 s.

Viruses were then grown in BHK-21 cells until a clear cytopathic effect was detected (when over 30% of cells started dying and detaching from the surface); the supernatant was collected and then centrifuged to remove cell debris, and virus titres were determined by plaque assays on BHK-21 cells seeded in 12-well plates. Plaque assays were conducted using 1.2% Avicel (FMC Biopolymer, London, UK) mixed 1:1 with media. Cells were fixed 72 h later with PFA and stained with 0.1% Toluidine Blue (Sigma Aldrich, St. Louis, MO, USA). PFU was calculated per ml. The lowest concentration detectable equates to 25 PFU/mL.

### 2.4. Virus Infections in Cells

Flasks of cells were maintained separately as 6 biological replicates per group. Cells from each flask were seeded at 5.5 × 10^5^ per well in 24-well plates and left to adhere overnight. Cells are around 80% confluency at time of infection. Cells were then infected with BUNV of an MOI of 0.1 in serum-free media. For 2HPCD (2-Hydroxypropyl-beta-cyclodextrin) experiments, cells were treated with PBS or 2HPCD (Merck, London, UK) which were added to the media at concentrations of 0.1 mM and 1 mM. Concentrations were selected based on previous work, showing that both concentrations are able to rescue Zika virus and DENV replication in *Wolbachia*-transinfected cells [11,12]. Cells were then incubated for 48 h prior to infection. Following 1 h after infection, media supplemented with FBS was added to the cells. After specified timepoints (24 h or 72 h p.i), the supernatant was collected and infectious viruses were assessed via a plaque assay; 500 μL of Trizol (Thermo Fisher, Altrincham, UK) was added to cells and RNA and DNA were extracted. Samples were stored at −80 °C until being processed.

### 2.5. Virus Infections of Mosquitoes

Four- to five-day-old females were provided with an infectious blood meal containing 1 × 10^7^ PFU/mL BUNV. For co-infection experiments, mosquitoes were fed an infectious blood meal containing 1 × 10^7^ PFU/mL BUNV and/or 1 × 10^7^ PFU/mL SFV4. Mosquitoes were collected 7, 9 or 12 days p.i. Mosquitoes were anaesthetized on ice, and their salivary glands were dissected and placed in serum-free GMEM media whilst the remaining carcass was collected in Trizol. All samples were stored in −80 °C until being processed. Tissue samples were then analysed via qRT-PCR for an analysis of the expression of BUNV using primers designed to bind to a part of the BUNV M (medium) segment or SFV E1, which have previously been established as a good indicator of the total viral RNA levels of each virus [29]. Salivary glands were analysed for viral titres via plaque assays.

### 2.6. RNA Purification and Quantification

All samples were lysed in 500 μL of Trizol (Qiagen, Manchester, UK) and tissue samples were homogenised using 1 mm glass beads on Percellys Homogeniser. A total of 0.1 mL of chloroform was then added to all samples and inverted 15 times to ensure mixing of solutions. Samples were then centrifuged at 12,000× *g* for 15 min at 4 °C in order to separate the mixture into a lower red phenol–chloroform phase and a colourless upper aqueous phase. The upper aqueous phase, containing the RNA, was transferred to a new tube containing an equal amount of isopropanol. Purified RNA was stored at −80 °C.

A High-capacity cDNA Reverse Transcription kit (Thermofisher) was used for cDNA conversion as per the manufacturer’s instructions. The final cDNA was then stored at −20 °C over the long term.

Total cDNA was diluted 1 in 5, using RNase-free water, and 1 μL was used per qPCR reaction in 384-well plates. A master mix was made up of primers, water, and Fast SYBR™ Green Master Mix (Applied Biosystems, Waltham, MA, USA) according to the manufacturer’s instructions. For each biological replicate, 3 technical replicates were made. A non-template control consisting of RNase-free water and master mix was also included. Samples were normalised to housekeeping gene RPS17 for virus quantification, whilst HTH (host homothorax) was used as a housekeeping gene measuring *Wolbachia* density. QPCR plates were run on the Applied Biosystems QuantStudio 5 flex machine. Melt curves were conducted to control for primer specificity. All samples were analysed by ΔΔct based on CT values calculated automatically by the QuantStudio software that detects the logarithmic phase of the PCR. Relative expression was then calculated. CT values higher than or equal to non-targeting controls were considered as negative.

Primers used: *Wolbachia* 16S F: GAAAGCCTGATCCAGCCATG, R: CGGAGTTAGCCAGGACTTCT; HTH F: TGGTCCTATATTGGCGAGCTA, R: TCGTTTTTGCAAGAAGGTCA; *Albopictus* RPS17 F: GAACGACAGCAGCGAAACTT, R: GTCACGAAACCAGCGATCTT; *Aedes* RPS17 F: CACTCCCAGGTCCGTGGTAT, R: GGACACTTCCGGCACGTAGT; BUNV M F: GGGGAAGATACAGGCAATGA, R: CCCACACACAGTCAGTAACAACA; SFV E1 F: CGCATCACCTTCTTTTGTG, R: CCAGACCACCCGAGATTTT.

### 2.7. Statistical Analysis

RT-qPCR data were analysed using Microsoft Excel by using the median of technical replicates normalised to the median of the technical replicates of the housekeeping genes. All data were analysed with GraphPad Prism 9 software. All datasets were tested for normal distribution prior to statistical tests. A nonparametric Kruskal–Wallis test was used for comparisons between more than two groups of non-normally distributed data, while nonparametric Mann–Whitney U was used for comparisons between two groups of non-normally distributed data. An ordinary ANOVA was performed for comparisons between more than two groups of normally distributed data, whilst a 2-way ANOVA was performed for more than two groups with multiple variables. Differences were considered significant at *p* < 0.05. Figures have statistical significance indicated as follows: * *p* < 0.05, ** *p* < 0.01, *** *p* < 0.001, **** *p* < 0.0001, and ns = not significant.

## 3. Results

### 3.1. Wolbachia Inhibits BUNV In Vitro and Infection Lowers Wolbachia Density

To determine whether *Wolbachia* is able to reduce BUNV replication in *Ae. Albopictus,* cells naturally carrying *w*AlbB, cells transinfected with either *w*Au or *w*Mel, and cells cured of *Wolbachia* were infected and viral titre and infectious particle levels measured.

**Figure 1:**
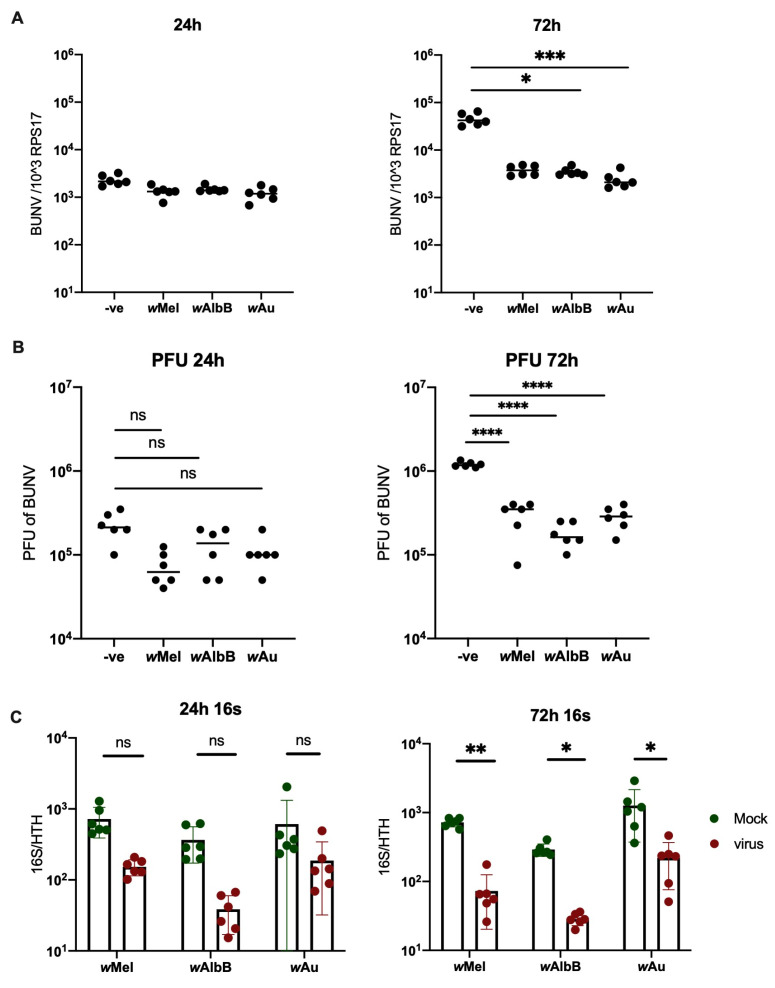
*Wolbachia* density is reduced by BUNV in Aa23 cells. Aa23 cells were infected with an MOI of 0.1. Samples were collected 24 h and 72 h p.i. N = 6 was used for each group. A line representing the sample mean has been included for each group. (**A**) A significant reduction in viral RNA in *Wolbachia* carrying lines can be observed at 72 h p.i. Cells were lysed in Trizol and analysed for viral RNA via qPCR. (**B**) Supernatant was collected and viral titres analysed via plaque assays. A significant reduction was observed in *Wolbachia*-carrying lines at 72 h p.i. (**C**) *Wolbachia* density of the cells was measured via qPCR. A significant reduction in density was observed in all three cell lines 72 h p.i. Bars represent the mean whilst error bars represent the standard deviation. * *p* < 0.05, ** *p* < 0.01, *** *p* < 0.001, **** *p* < 0.0001, ns = not significant.

The presence of *Wolbachia* in Aa23 cells persistently reduced BUNV infection at 72 h.p.i (Figure 1A,B). Whilst a drop in viral titres could initially be observed at 24 h.p.i, this was not significant. However, virus infection was not blocked completely. Instead, viral RNA dropped by more than a log-fold at 72 h, whilst viral titres analysed via plaque assays exhibited slightly less than a log-fold drop. Interestingly, when quantifying the *Wolbachia* density of all cell lines used, we observed that BUNV reduces *Wolbachia* density in vitro (Figure 1C). This does not occur when *Wolbachia*-carrying Aa23 cells are infected with +ssRNA viruses [25].

**Figure 2:**
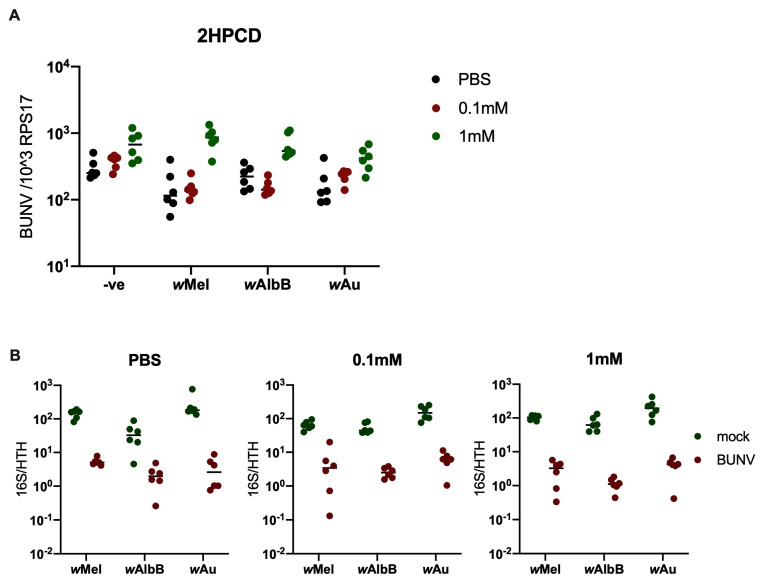
2HPCD rescues BUNV infection in Aa23 cells. Aa23 cells were pre-treated with PBS, 0.1 mM or 1 mM of 2HPCD for 48 h prior to infection with an MOI of 0.1 of BUNV. N = 6 was used for each group. Samples were collected 24 h p.i and analysed via qPCR. A line representing the sample mean has been included for each group. (**A**) BUNV infection in cells is rescued when 1 mM 2HPCD was added. (**B**) *Wolbachia* density in cells was not rescued by the addition of 2HPCD.

As 2HPCD has been demonstrated to partially rescue *w*Mel-mediated antiviral inhibition against flavivirus infection but not alphavirus infection, this study also investigated the impact of 2HPCD on BUNV infection. As BUNV has previously been shown to require cholesterol during endosomal escape, it was hypothesised that *Wolbachia* perturbation of cellular cholesterol is responsible for inhibiting BUNV infection [30]. To test this hypothesis, we treated Aa23 cells with 2HPCD, and rescue of BUNV infection in *w*Mel, *w*AlbB, and *w*Au cells was observed (Figure 2). However, 2HPCD also enhanced infection in the non-*Wolbachia*-carrying control cells, suggesting that the additional cholesterol boosts BUNV infection (although not significantly). When looking at *Wolbachia* density, no difference was observed when 2HPCD was added (Figure 2B). This means that the density reduction observed in the presence of BUNV is not rescued by 2HPCD.

### 3.2. BUNV Is Not Inhibited by Wolbachia In Vivo but Infection Does Lower Wolbachia Density

**Figure 3:**
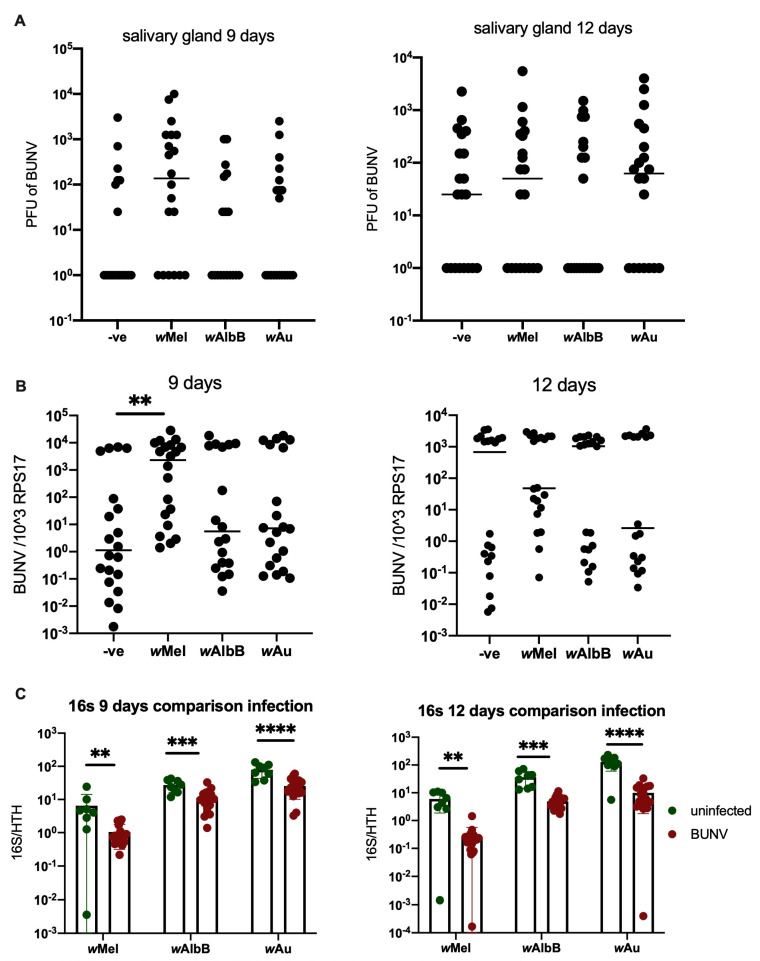
*Wolbachia* does not block BUNV infection in *Ae. aegypti* mosquitoes. *Ae. aegypti* mosquitoes were infected via infectious blood meal containing 1 × 10^7^ PFU/mL of BUNV. Salivary glands and remaining carcass were collected at 9 and 12 days p.i. Each group has N = 20 with the exception for *w*AlbB at 9 days which has N = 18. A line representing the sample mean has been included for each group. (**A**) Salivary glands were placed in 250 uL serum free media and viral titres measured via plaque assays. (**B**) Carcass stored in 500 uL Trizol was analysed via qPCR. (**C**) *Wolbachia* density was measured by quantifying 16S via qPCR on mosquito carcass. Bars represent the mean whilst error bars represent the standard deviation. ** *p* < 0.01, *** *p* < 0.001, **** *p* < 0.0001, ns = not significant.

Previous research investigating the interactions between *Wolbachia* and -ssRNA viruses has primarily studied these interactions in vitro in cell lines. However, in order to study these interactions in a more natural infection model, the effect of *Wolbachia* on BUNV infection in vivo was investigated. This was achieved using four *Ae. aegypti* mosquito lines—LS (*Wolbachia*-free), *w*Mel, *w*AlbB and *w*Au—all of which were infected with BUNV following feeding with infectious blood meal. Samples were collected at 7, 9 and 12 days post infection (p.i) and they exhibited no difference in virus quantity, viral RNA and viral titres between the groups at either timepoint (see Figure 3), with the exception of *w*Mel, where an increase in viral RNA was observed at 9 days p.i. This shows that BUNV is not blocked by *Wolbachia* in vivo. At 7 days, a low percentage of BUNV-positive mosquito salivary glands was observed in all groups (see Figure S1). Interestingly, *Wolbachia* density was reduced in vivo following BUNV infection (see Figure 3C). More than a log-fold reduction in density was observed at 12 days p.i, a reduction similar to that observed in cells. This density reduction could partially explain why BUNV infection is not successfully blocked by *Wolbachia*.

**Figure 4:**
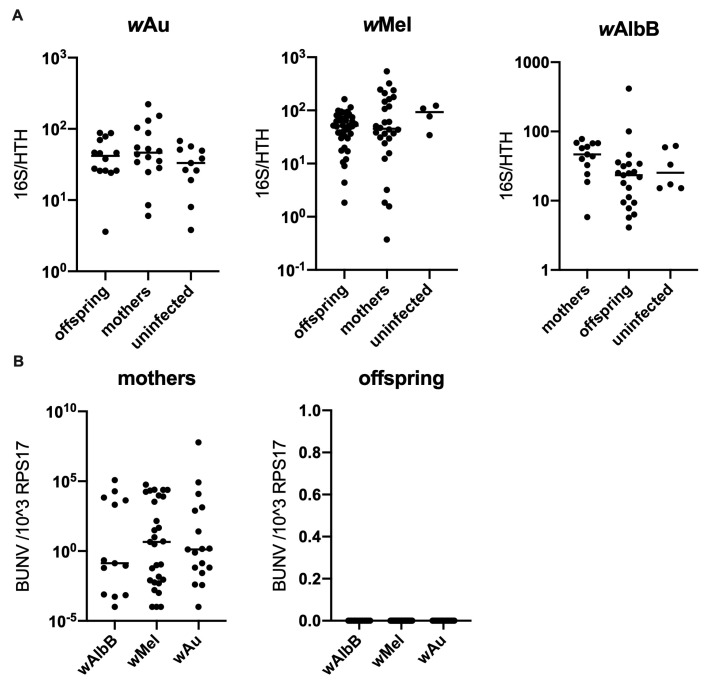
*Ae. aegypti* mosquitoes were infected with BUNV following feeding on an infectious blood meal containing 1 × 10^7^ PFU/mL of BUNV. Bloodfed mosquitoes were individualised and allowed to lay eggs. Eggs were collected and, following drying, they were hatched. Adult mosquitoes were collected 4–6 days post emergence. Both mothers and offspring were collected and placed into Trizol and analysed by qPCR. A line representing the sample mean has been included for each group. (**A**) *Wolbachia* density did not change between the groups. (**B**) BUNV was detected in the mothers but not the offspring.

As a reduction in *Wolbachia* density following BUNV infection was observed, experiments were carried out to determine if this reduction led to a reduction in transgenerational levels of *Wolbachia*. Eggs laid by mosquitoes infected with BUNV via an infectious blood meal were therefore collected. Egg cones were then dried and hatched. Adult mosquitoes were collected 3 days post emergence and RNA and DNA extractions were conducted on whole mosquitoes. Measuring of 16S demonstrated that BUNV does not reduce *Wolbachia* density in future generations (see Figure 4A). In addition, no BUNV was detected in the offspring of BUNV-infected mothers, suggesting that BUNV does not undergo transovarial transmission (from mother to offspring). This could also help to explain why no effect on *Wolbachia* density is observed in later generations.

### 3.3. Co-Infection of BUNV and SFV Leads to a Significant Decrease in SFV Levels

**Figure 5:**
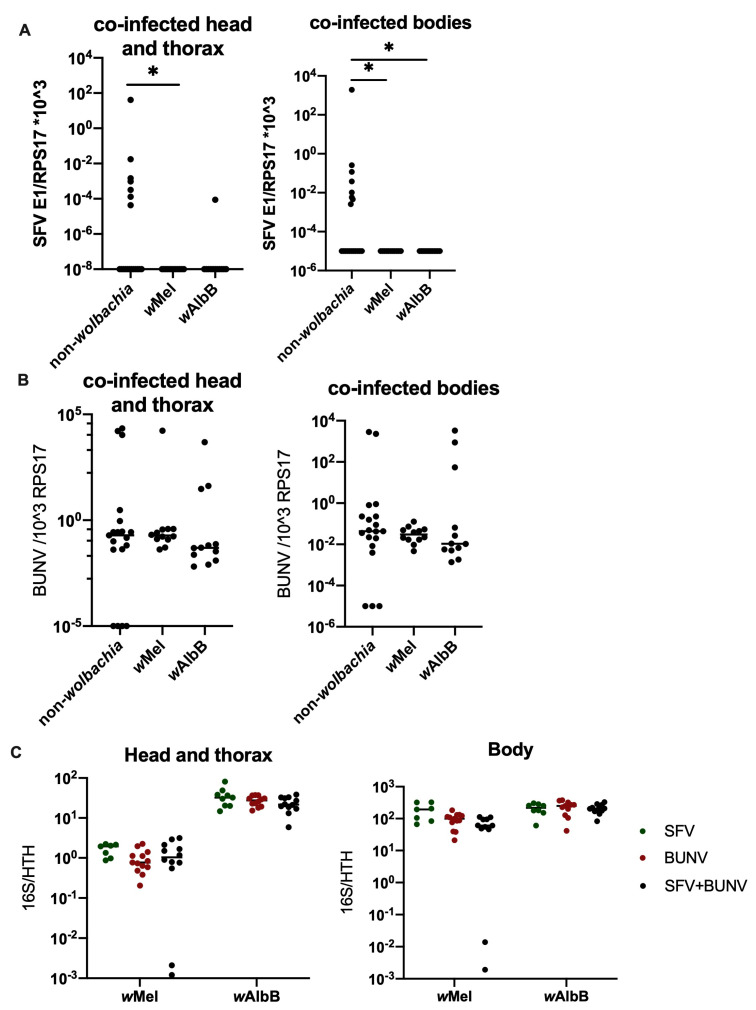
*Ae. aegypti* mosquitoes were infected with BUNV and/or SFV following feeding on an infectious blood meal containing 1 × 10^7^ PFU/mL of both viruses. Bloodfed mosquitoes were collected 7 d.p.i. Heads and thorax were separated from body and these samples were processed separately via qPCR. A line representing the sample mean has been included for each group. (**A**) SFV infection in the co-infected mosquitoes was quantified in the head and thorax and the bodies via qPCR. (**B**) BUNV infection in the co-infected mosquitoes was quantified sin the head and thorax and the bodies via qPCR. (**C**) *w*Mel and *w*AlbB density of SFV only, BUNV only, or co-infected mosquitoes was quantified via qPCR in head and thorax and bodies. * *p* < 0.05.

**Figure 6:**
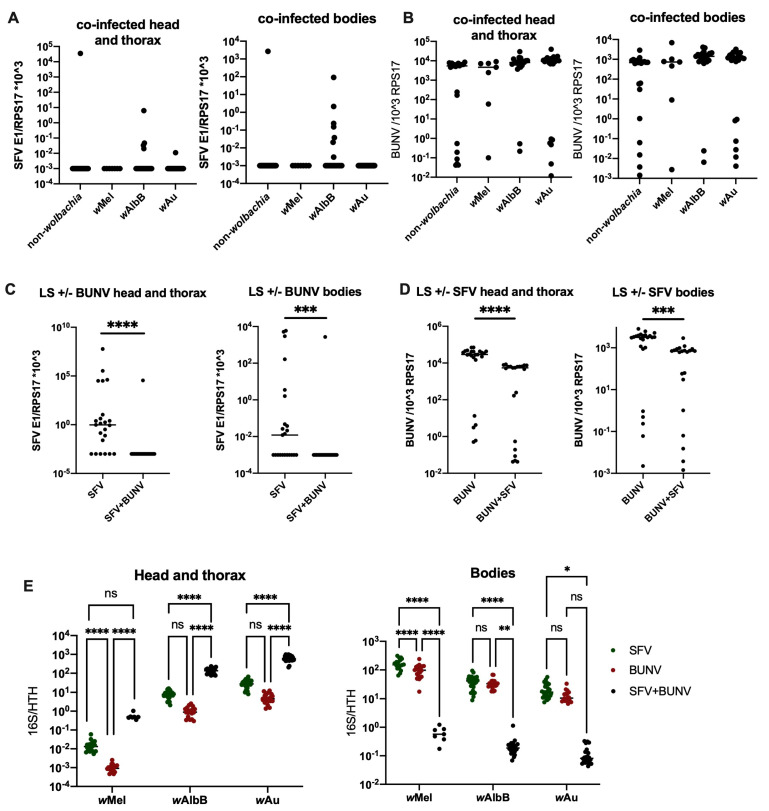
*Ae. aegypti* mosquitoes were infected with BUNV and/or SFV following feeding on an infectious blood meal containing 1 × 10^7^ PFU/mL of both viruses. Bloodfed mosquitoes were collected 12 d.p.i. Heads and thorax were separated from body and these samples were processed separately via qPCR. (**A**) SFV infection in the head and thorax and the bodies of co-infected mosquitoes (**B**) BUNV infection in the head and thorax and the bodies of co-infected mosquitoes (**C**) SFV infection in non-*Wolbachia*-carrying mosquitoes infected with SFV only or alongside BUNV (**D**) BUNV infection in non-*Wolbachia-*carrying mosquitoes infected with BUNV only or alongside SFV (**E**) *Wolbachia* density in the head and thorax and bodies. * *p* < 0.05, ** *p* < 0.01, *** *p* < 0.001, **** *p* < 0.0001, ns = not significant.

To assess the potential impact that a reduction in *Wolbachia* density may have in cases where co-infection of BUNV with a +ssRNA virus has occurred, *Ae. aegypti* mosquitoes were co-infected with both Semliki Forest Virus (SFV) and BUNV via an infectious blood meal containing 1 × 10^7^ PFU/mL of each virus. Interestingly, *Wolbachia* was still able to successfully block SFV infection during co-infection at 7 days p.i (Figure 5). At 12 days p.i, *Wolbachia* blocked SFV in both single- and co-infected mosquitoes, and interestingly, SFV infection was almost completely blocked in LS as well (Figure 6A,C and Figure S2A). No impact on BUNV infection was observed in either single- or co-infected *Wolbachia*-carrying lines, but a slight drop in BUNV titre was observed in the co-infected LS mosquitoes compared to mosquitoes infected with BUNV on its own (Figure 6B,D and Figure S2A). *Wolbachia* density of all strains was significantly reduced in the mosquito bodies when co-infected with BUNV and SFV as opposed to infection with one virus at a time, but not in the head and thorax and only at 12 d.p.i. Instead, in the head and thorax, the *Wolbachia* density of all strains was significantly down in the mosquitoes infected with BUNV. Interestingly, *Wolbachia* density in the head and thorax was also slightly lower in SFV-only-infected mosquitoes as compared to co-infected mosquitoes.

## 4. Discussion

This study demonstrates, for the first time, a negative impact on *Wolbachia* density by—ssRNA virus Bunyamwera. The data presented here suggest that *Wolbachia* reduces BUNV infection in Aa23 cells as well as in *Ae. aegypti* mosquitoes. Whilst future generations did not appear to be impacted, it remains unknown whether a secondary BUNV infection or eggs laid later following a second blood feed may be impacted.

Interestingly, a reduction in BUNV infection was observed in Aa23 cells in vitro in the presence of *Wolbachia.* It remains unclear why this reduction is not observed in vivo as both models exhibit a reduction in *Wolbachia* density. Virus infection is not blocked completely within the cells; BUNV was merely reduced and given variations in *Wolbachia* density between tissues. It is possible that the negative impact on the virus is insufficient to reduce overall infection levels within the whole mosquito. It is also possible that the observed differences are due to the *Aedes* species, as Aa23 cells are *Ae. albopictus* and in vivo experiments were carried out in *Ae. aegypti.* However, a previous study investigating the ability of BUNV to replicate in *Ae. albopictus* and *Ae. aegypti* cells found that the virus replicates to a similar extent in both cell types during acute infection [31].

As *Wolbachia w*Mel and *w*AlbB, but not *w*Au, have been found to cause perturbations in intracellular cholesterol/lipid trafficking, we wanted to investigate whether the release of trapped cholesterol was sufficient to prevent a reduction in BUNV infection in cells. Indeed, when 2HPCD was added to cells, we no longer observed a reduction in BUNV infection. It is possible that the reduction in *Wolbachia* density is caused by competition for resources within the cells when both *Wolbachia* and BUNV are present. In this study, it was determined that this reduction is not caused by lack of cholesterol. Further studies are required to determine the mechanism involved in BUNV-mediated *Wolbachia* density reduction.

The data presented here show that the initial reduction in *Wolbachia* density following BUNV infection does not persist in future generations. This means that *Wolbachia*-carrying mosquitoes laid by mothers infected with BUNV via an infectious blood meal are not compromised in their ability to block other +ssRNA arbovirus infections. The lack of impact on future generations observed in this study is likely to be due to the early timepoint studied, as eggs are laid a few days following infection. This could be insufficient time for the virus to have an impact on *Wolbachia* density in mothers before egg laying. In fact, when looking at earlier infectious timepoints (9 days p.i) in Figure 3, we observe a higher *Wolbachia* density in BUNV-infected mosquitoes than at 12 days p.i. Similarly, for the co-infection experiments, we observed no impact on *Wolbachia* density at 7 days p.i in comparison to 12 days p.i (Figure 5C and Figure 6E, respectively). This suggests that there is a gradual reduction in *Wolbachia* density following infection, and thus, when the eggs were laid 3–5 days p.i, the *Wolbachia* density within the mosquito might not have reduced enough to influence density in subsequent generations. It is possible that eggs laid in subsequent gonotrophic cycles or at later timepoints following BUNV infection could exhibit a reduced *Wolbachia* density. In addition, it is possible that mosquitoes infected with BUNV via alternate transmission routes other than blood feeding, e.g., venereal transmission, could give rise to offspring exhibiting a reduction in *Wolbachia* density.

Similarly, these data showed that co-infection with SFV and BUNV in *Wolbachia*-carrying *Ae. aegypti* mosquitoes does not prevent blocking of SFV at 7 days p.i (Figure 5). At 12 days p.i, there was instead a reduction in SFV infection observed in co-infected LS mosquitoes. This appears to be due to competition between the viruses, as this phenomenon was observed in non-*Wolbachia*-carrying mosquitoes infected with SFV with or without BUNV (Figure 6C). BUNV titres were also significantly reduced, although to a lesser extent than observed with SFV (Figure 6D). Similarly, during co-infection of SFV and BUNV, *Wolbachia* densities of all strains were significantly down-regulated in the mosquito bodies but not in the head and thorax. It is likely that this phenomenon is due to competition for resources between the two viruses and the endosymbiont *Wolbachia*. It remains unknown, however, whether a secondary infection occurring at a later timepoint following BUNV infection would have an impact. The timing and severity of effect on *Wolbachia* density are likely to play a crucial role in this. Future studies will be required to further assess the potential impact that -ssRNA virus infections and other virus co-infections can have on *Wolbachia* density and *Wolbachia-*mediated virus blocking.

It is crucial to understand the impact that future -ssRNA virus outbreaks can have on *Wolbachia* release strategies. It is possible that such outbreaks could compromise population replacement efforts as well as *Wolbachia’s* ability to block medically relevant arboviruses such as DENV. Further studies looking at other *Wolbachia* strains would also be beneficial, as these interactions involving *Wolbachia* density reduction may prove to be specific to particular strains. Whilst BUNV is not widespread globally, there are many other viruses belonging to the Bunyavirales order that may potentially cause a similar drop in *Wolbachia* density following infection, including Rift Valley fever virus. This could also be the case for the wide range of ISVs, which have the potential to have a much bigger impact on *Wolbachia* dynamics given their higher prevalence and ability to transmit vertically [32,33]. Such viruses include Phasi Chareon virus, Badu virus and Gouléako virus [34,35,36,37]. As ISVs remain understudied, there are likely to be many undiscovered viruses that have the potential to impact *Wolbachia* density in *Aedes* field populations. Further research is required to determine the impact other that Bunyaviridae and virus co-infections have on *Wolbachia* density, including ISVs. Deepening our understanding will help optimise *Wolbachia* field releases and enable targeted solutions to be implemented in a timely manner in response to future outbreaks.

## Data Availability

All raw data can be accessed on the Glasgow University repository (DOI to follow).

## Supplementary Materials

The following supporting information can be downloaded at https://www.mdpi.com/article/10.3390/v16081336/s1.
