# Supplementary material for: Bunyamwera Virus Infection of Wolbachia-Carrying Aedes aegypti Mosquitoes Reduces Wolbachia Density
Source: Viruses. 2024 Aug 21;16(8):1336. doi: 10.3390/v16081336 (PMC11360823; doi:10.3390/v16081336)
Supplement: Supplementary file 1 [file viruses-16-01336-s001.zip › viruses-3103397-supplementary.pdf]

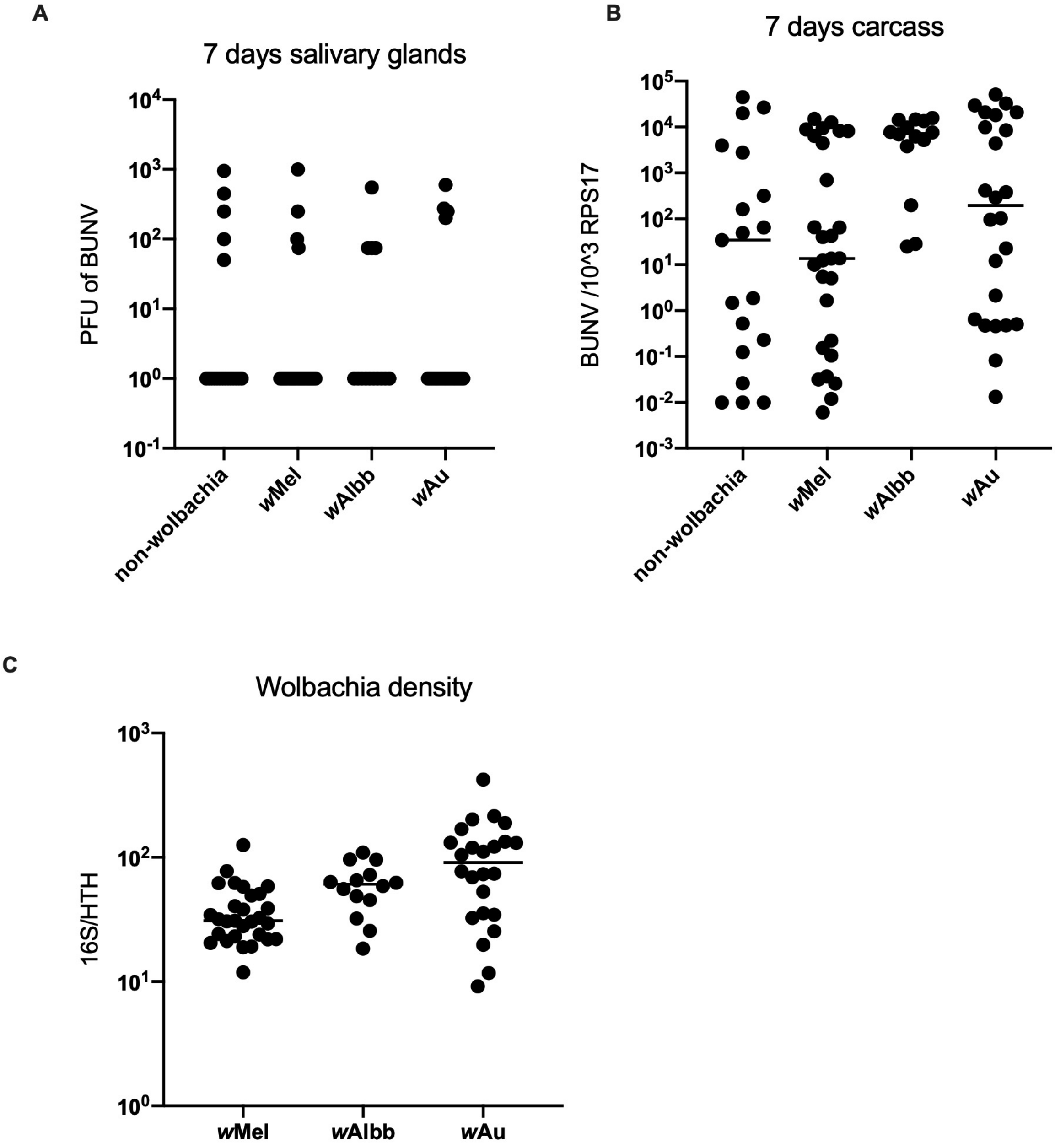

sFigure 1: *Wolbachia* doesnt block BUNV infection in Ae. aegypti mosquitoes. *Ae. aegypti* mosquitoes were infected via infectious blood meal containing 1x10^7 PFU/ml of BUNV. Salivary glands and remaining carcass was collected at 7 days p.i. Each group has between 14 and 29 replicates. **A**. Salivary glands were placed in 250ul serum free media and viral titres measured via plaque assays. **B**. Carcass stored in 500ul trizol was analysed via qPCR. **C**. *Wolbachia* density was measured by quantifying 16S via qPCR on mosquito carcass.

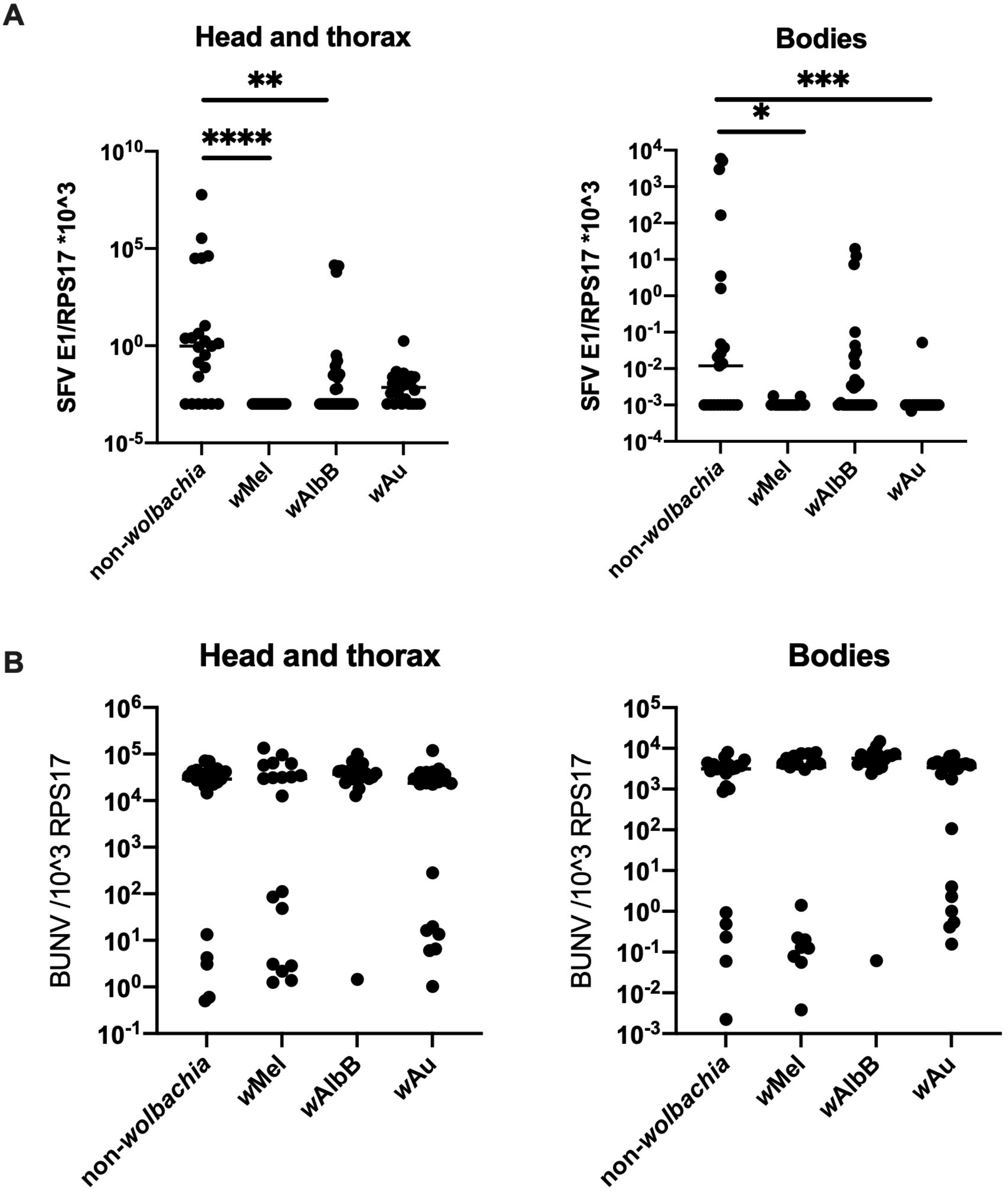

sFigure 2: *Ae. aegypti* mosquites were infected with BUNV and/or SFV following feeding on an infectious blood meal containing $1x10^7$ PFU/ml of both viruses. Bloodfed mosquitoes were collected 12 d.p.i. Heads and thorax was seperated from body and these samples were processed seperately via qPCR. A. SFV quantity of mosquitoes infected only with SFV. B. BUNV quantity of mosquitoes infected only with BUNV.
